# Experimental Assessment of Corrosion Properties for Materials Intended for Heavy Crude Processing

**DOI:** 10.3390/ma17215275

**Published:** 2024-10-30

**Authors:** Raúl González-Durán, Alvaro Rodríguez-Prieto, Ana María Camacho, Darío Y. Peña-Ballesteros, Aníbal Serna

**Affiliations:** 1 Department of Manufacturing Engineering, UNED, 28040 Madrid, Spain; alvaro.rodriguez@ind.uned.es (A.R.-P.); amcamacho@ind.uned.es (A.M.C.); 2 Department of Materials Science and Metallurgical Engineering, Universidad Industrial de Santander, Bucaramanga 680003, Colombia; dypena@uis.edu.co; 3 Department of Metallurgical Engineering, UPTC, Tunja 47215, Colombia; anibalsernag@gmail.com

**Keywords:** total acidity number (TAN), sulfur content, damage mechanisms, materials performance, corrosion

## Abstract

Heavy crude oil processing presents significant challenges owing to its complex composition and requirement for processing conditions, which increase the process safety risk in crude processing units, such as fixed equipment, for instance pressure vessels and pipes. The aim of this work is to evaluate the influence of heavy crude oils named A and B and the effect of sulfur-rich compounds and organic acids on the performance at high temperatures of three metallic alloys (5Cr-1/2Mo/ASTM A335GP5, X6CrNiMoTi17122/AISI-SAE 316Ti and Ni66.5Cu31.5/Monel 400) and propose an alternative to be used in pressure vessels and piping in refineries. This work was based on the need to understand the corrosivity of two heavy crude oils (A and B) from eastern Colombia in three materials, evaluated at three temperatures (200 °C, 250 °C and 300 °C) under the same conditions of pressure (200 psi) and rotation velocity (600 rpm) in a dynamic autoclave to simulate atmospheric conditions and conditions in vacuum refinery towers. An understanding of how these factors interact with the fundamental principles of corrosion kinetics is essential for developing an effective corrosion mitigation strategy. The results were interesting for applications requiring high corrosion resistance. X6CrNiMoTi17122/AISI-SAE 316Ti is a solid candidate for this application, with corrosion rates of 0.2 to 0.87 mpy. Ni66.5Cu31.5/Monel 400 exhibited significant corrosion rates up to 74.89 mpy, especially at higher temperatures (300 °C). 5Cr-1/2Mo/ASTM A335GP5 showed a generally moderate corrosion rate (2.04–5.57 mpy) in the evaluated temperature range.

## 1. Introduction

The demand for crude oil as an energy source is continuously increasing, driven by an ever-growing population and economic development [1]. In recent years, Latin America has faced a series of changes in energy security as a result of energy consumption in the region caused by growth and economic development [2]. Therefore, energy security and independence are priorities for American nations.

The increase in energy demand makes it necessary to diversify energy sources to guarantee energy security and sovereignty since the region’s light oil and gas deposits will continue to be depleted. Consequently, as long as fossil fuels remain an important part of the energy mix, the industry must make significant efforts to ensure the sustainability, efficiency and reliability of current processing plants, such as oil refineries and petrochemical plants, to achieve this. Colombia’s UPME (Unidad de Planeación Minero-Energética) predicts measures to enhance oil supply security, primarily through increased refining capacity [3]. Additionally, Colombia’s national oil company is focusing on increasing the performance of the maturing Rubiales, Castilla, Jazmin and Chichimene fields through infill drilling, which make up the main source of heavy crude oil production in Colombia and constitute its main export pillar, rather than undertaking new exploration in the Llanos region. At the moment, the total crude oil refining capacity in Colombian refineries is 378,600 barrels per day. The refineries were originally built to process light, sweet crude oil from fields such as Cusiana and Cupiagua, but Colombia’s most important oil blend is the Castilla Blend, a heavy and sour crude oil with a high sulfur content (S ≈ 1.97%) and an API gravity of 18.8 degrees. Therefore, the growing production of heavy crude oil in Colombia has presented challenges for the refining and transportation sectors [4].

The processing of heavy crude oils in refineries, especially in existing refineries, presents many difficulties due to their unfavorable characteristics and the low quality of the crude oil. The refining processes face enormous challenges, such as general corrosion problems in fixed equipment. Other damage mechanisms, such as fouling in heat exchangers, lead to increased operating and maintenance costs as well as potential production losses [1].

In atmospheric and vacuum distillation columns operating in refineries, sulfur and naphthenic acid corrosion share a wide range of occurrence temperatures [5]. Also, naphthenic acid corrosion occurs primarily in the high-velocity area of a crude distillation unit at temperatures ranging from 220 to 400 °C [6]. Sulfidic corrosion occurs in metals and alloys due to the pernicious effect of sulfur compounds at high temperatures, usually in the range of 230–540 °C [6]. Furthermore, several authors have proposed that the corrosion rate is affected by the sulfur and naphthenic acid concentrations in crude oil [6,7].

**Figure 1:**
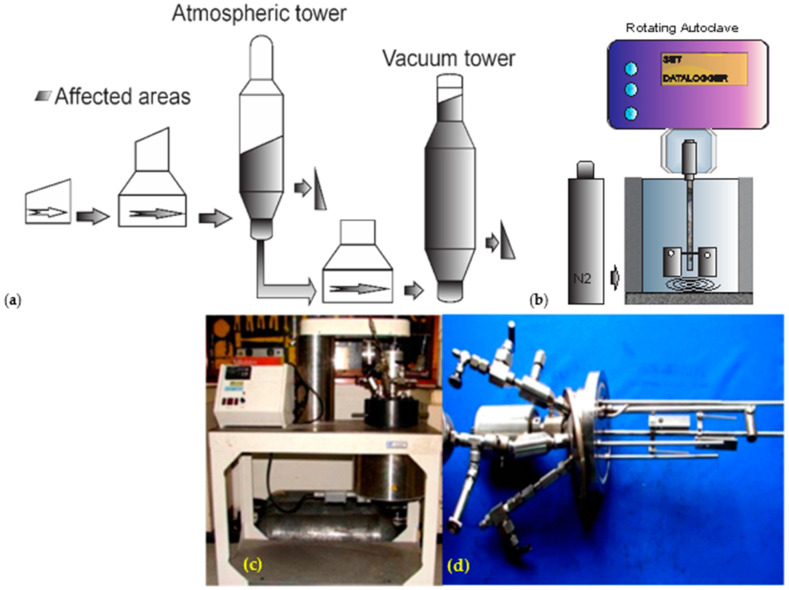
(**a**) Refinery corrosion-affected areas. (**b**) Corrosion testing circuit apparatus, coupons and setting. (**c**) Corrosion testing apparatus. (**d**) Coupons rag setting.

Heavy crude oil composition includes the sulfur compounds elemental sulfur (S), hydrogen sulfide (H_2_S), mercaptans, organic sulfides, disulfides and polysulfides, carboxylic acids (including naphthenic acids) and metals, among others [6,8]. Usually, the corrosiveness of a crude oil is defined by its total acid number (TAN mg KOH/g), and total sulfur content [6]. Neither TAN value nor total sulfur content alone is sufficient to define the corrosiveness of crudes [6]. Nevertheless, they can cause corrosion under refinery processing conditions at elevated temperatures > 190 °C (naphthenic acids) and >230 °C (sulfur compounds) [6]. Sulfidic corrosion is a kind of corrosion of metals and alloys by sulfur compounds at high temperatures, occurring in crude distillation (atmospheric and vacuum), hydro-processing, catalytic reforming and coking units and in fractionation and distillation facilities downstream from hydrotreaters and hydrocrackers. The affected areas are shown in Figure 1a. Naphthenic acid corrosion occurs only at high temperatures (190 °C < T < 360 °C), sulfidic corrosion proceeds more intensively in the presence of H_2_, and inhibition of naphthenic acid corrosion is caused by sulfide scale, which forms in the presence of H_2_S [6]. The weight loss (WL) method to determine the corrosiveness of crudes has prolonged exposure time and low reproducibility, and coupons (used in the WL method) can sometimes increase weight due to adhesive product buildup. Nevertheless, there was no correlation between certain physicochemical properties of crudes (TAN, sulfur and water content) and corrosiveness [6]. When naphthenic acid corrosion and sulfidic corrosion occur together, they interact and can be described (Equations (1)–(3)) by the following reactions [5,9,10]:

Fe + 2RCOOH ⇄ Fe (RCOO)_2_ + H_2_ (1)

Fe + H_2_S ⇄ FeS + H_2_ (2)

Fe (RCOO)_2_ + H_2_S ⇄ FeS + 2RCOOH (3)

These chemical equations (Equations (1)–(3)) summarize the combined corrosive reactions of sulfur and naphthenic acid compounds. Naphthenic acid attacks the metal (Equation (1)), forming iron naphthenates that are oil soluble and can be entrained by the flow [11]. Simultaneously, some sulfur-containing compounds decompose at high temperatures, generating hydrogen sulfide (H_2_S) that reacts with the metal (Equation (2)) and forms iron sulfide (FeS) [12]. FeS is insoluble in oil and deposits on the metal surface, offering some limited protection against naphthenic acid attack [11]. According to Equation (3), the hydrogen sulfide reacts with the soluble iron naphthenates, regenerating the naphthenic acids and generating more FeS [5,10,11].

Depending on the presence of a sulfide surface layer, naphthenic acid corrosion may be classified into three types [13]:

- Pure naphthenic acid corrosion with no or negligible effect caused by sulfur compounds.
- Sulfidation corrosion, which is accelerated corrosion due to the destruction of a sulfide surface layer by naphthenic acid.
- Inhibited naphthenic acid corrosion, which is the inhibition of naphthenic acid corrosion due to the presence of a sulfide surface layer.

The corrosion mechanism due to naphthenic acid and sulfur has been intensively studied. Nonetheless, the mechanism associated with these species is still not clearly understood, although the effects of factors such as Sulfur/TAN ratio, temperature and the molecular weights and structures of the corrosive species present in crude oil have been considered key aspects to understand the interaction between naphthenic acid and sulfur compounds and their effects [9].

Refinery engineers must adopt special strategies for mitigating acidic crude oil corrosivity effects by modifying the refinery unit’s design and procedures and by opting for better materials for the critical components. To make these kinds of decisions with more confidence, it is important to improve our understanding of the mechanism of naphthenic acid corrosion and its interaction with sulfidic corrosion [5].

Corrosion coupons (5Cr-1/2Mo/ASTM A335GP5, steel, X6CrNiMoTi17122/AISI-SAE 316Ti and Ni66.5Cu31.5/Monel 400) were evaluated in two crudes (A and B) at three temperatures (200 °C, 250 °C and 300 °C) in the same conditions of pressure (200 psi) and rotation velocity (600 rpm) in a dynamic autoclave to simulate atmospheric conditions and conditions in vacuum refinery towers.

This work is based on the need to understand the corrosive effect of processing heavy crude oil from eastern Colombia in the national refineries’ vacuum and topping processes by dynamic autoclave simulation at high temperature.

This study aimed to evaluate the corrosive effect of two Colombian heavy crude oils, crude A (TAN: 7.7, sulfur: 1.5) and crude B (TAN: 0.1, sulfur: 2.3%), on three different materials: 5Cr-1/2Mo steel (ASTM A335GP5), AISI 316Ti stainless steel and Nickel 60% alloy (Monel 400). The materials were exposed to the crude oils at various temperatures (200 °C, 250 °C and 300 °C) in a dynamic autoclave under controlled conditions (pressure: 200 psi, rotation velocity: 600 rpm) to simulate refinery tower conditions. 

## 2. Materials and Methods

In this study, three metallic alloys were selected: 5Cr-1/2Mo/ASTM A335GP5 (ferritic steel), X6CrNiMoTi17122/AISI-SAE 316Ti (austenitic stainless steel) and the Ni66.5Cu31.5/Monel 400 (nickel-copper alloy), due to the potential good response to the required operating conditions in the heavy crude processing industry. Through gravimetric testing in heavy crude oil and dynamic autoclave evaluations at elevated temperatures, these alloys were evaluated under demanding conditions. Corrosion coupons with dimensions of 18 cm^2^, previously weighed, were used to characterize the alloys chemically and physically after exposure.

First, the coupons were visually inspected. Then, the corrosion deposits were characterized using scanning electron microscopy (SEM) and their composition was analyzed by energy-dispersive X-ray spectroscopy (EDS) and X-ray diffraction (XRD).

The 5Cr-1/2Mo/ASTM A335GP5 steel was selected as a typical material in topping units and vacuum towers. The X6CrNiMoTi17122/AISI-SAE 316Ti steel and Ni66.5Cu31.5/Monel 400 alloy were selected as novel alternative materials with the goal of achieving enhanced corrosion resistance in the naphthenic acid and sulfidic environments commonly found in processing units. Heavy crude oils selected for testing were obtained from the fields, characterized (TAN: ASTM D 664 [14]; S%: ASTM D 4294 [15]), and designated as crude A (TAN: 7.7, sulfur: 1.5) and crude B (TAN: 0.1, sulfur: 2.3%), the characteristics of typical heavy crude oils processed in Colombian refineries [14,15].

The experimental methodology to evaluate corrosion resistance consists of the preparation of a corrosion circuit with a rotative autoclave type batch and the process safety system shown in Figure 1b–d.

Prior to testing, the corrosion coupons (5Cr-1/2Mo/ASTM A335GP5 ferritic steel, X6CrNiMoTi17122/AISI-SAE 316Ti austenitic stainless steel and Ni66.5Cu31.5/Monel 400) were thoroughly cleaned, weighed, and introduced into a rotating autoclave. This autoclave was specifically configured to operate at temperature (temperature range of 200, 250 or 300 °C, velocity 600 rpm, pressure 200 psi and time 96 h), thereby replicating the conditions typically found in refinery environments (see Table 1 for detailed specifications). Following the test, the coupon pairs were weighed and subjected to microscopic examination. This examination allowed for a comprehensive assessment of both the substrate and any potential scale layers that might have formed. It is imperative that the materials are consistently referred to using the designation criteria established at the beginning of the paper. The scale layers were evaluated by scanning electron microscopy (SEM), energy-dispersive X-ray spectroscopy (EDS) and X-ray diffraction (XRD) to identify the composition and morphological structure of any scales. The coupon was cleaned to white metal, then weighted and the corrosion rate calculated according to NACE RP 0775 [16] and ASTM G1 [17].

Table 2 describes the crude oil characterization results and Table 3 describes the material composition.

## 3. Results

### 3.1. Corrosion Rate Evaluation

**Figure 2:**
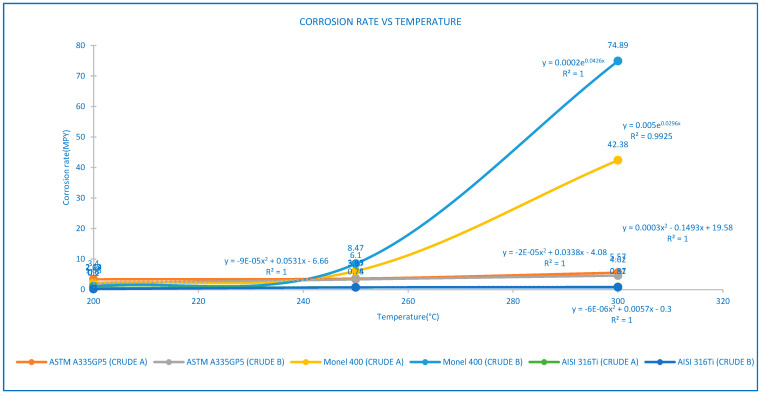
Corrosion rate vs. temperature results at test time (96 h), regression mathematical model.

The corrosion rate of the metallic coupons was measured by analyzing the weight loss data after a 96 h corrosion test under dynamic conditions (600 rpm) in the same conditions of pressure (200 psi) at high temperature (200–300 °C), using the equation for corrosion rate in Standard NACE RP 0775 [16], and the results are provided in Figure 2.

This study evaluates the corrosiveness of 5Cr-1/2Mo/ASTM A335GP5 (ferritic steel), X6CrNiMoTi17122/AISI-SAE 316Ti (austenitic stainless steel) and the Ni66.5Cu31.5/Monel 400 (nickel-copper alloy) by calculating mass loss and corrosion rate. Coupon mass loss was determined according to ASTM G1 [17]. The corrosion rate (TC) in mils per year (Mpy) was calculated using Equation (4) as follows:

(4) CR=(K·W)/(A·T·D)

where:

*CR*: Corrosion rate (mpy)

*K*: 3.45 × 10^6^

*W*: Mass loss (g)

*A*: Coupon area (cm^2^). The coupon area was 18 cm^2^

*T*: Exposure time (hours)

*D*: Density of the material (g/cm^3^)*

The densities used for the different materials (5Cr-1/2Mo steel: 7.86 g/cm^3^, 316Ti stainless steel: 7.98 g/cm^3^, Monel 400: 8.80 g/cm^3^) are necessary for calculating the corrosion rate according to the equation mentioned above [17].

Figure 2 describes the general trend of results of the corrosion rate (mpy) vs. temperature (°C) for three alloys, 5Cr-1/2Mo/ASTM A335GP5, X6CrNiMoTi17122/AISI-SAE 316Ti steels and Ni66.5Cu31.5/Monel 400 evaluated in crude A and crude B at three temperatures (200 °C, 250 °C and 300 °C).

In the case of 5Cr-1/2Mo/ASTM A335GP5 steel for heavy crude oils A and B, the graph indicates that corrosion rate increased gradually with increasing temperature (200–250 °C), with a greater increase in corrosion rates being observed at a temperature of 300 °C for crude A and crude B and corresponding to a polynomial adjustment and regression mathematical model with R^2^ equal to 1 for crude A and crude B. It was observed that the corrosion rates of crude A at the three temperatures analyzed were greater than those of crude B.

Regarding X6CrNiMoTi17122/AISI-SAE 316Ti, for heavy crude oils A and B, the chart describes a slight increase in the corrosion rate with increasing temperature (200–300 °C), which corresponds to a polynomial adjustment and regression mathematical model with R^2^ equal to 1 for crudes A and B. The chart graph describes a slight increase in the corrosion rate with temperature (200–300 °C) for heavy crude oils A and B. The corrosion rates for crude A were greater than those for crude B for the three temperatures.

The greatest increase in corrosion rate (crude A and B) was for Ni66.5Cu31.5/Monel 400, associated with increasing temperature (200–300 °C), and matching an exponential adjustment and regression mathematical model with R^2^ values of 1 and 0.99 for crude A and crude B, respectively. When comparing crudes A and B, corrosion rates for crude B at 200 °C were slightly lower than those for crude A. In contrast, the corrosion rate was higher for crude B at temperatures of 250–300 °C.

The highest difference in corrosion rates was for the Ni66.5Cu31.5/Monel 400 alloy evaluated in crude A and crude B for temperatures 250 °C and 300 °C, which experienced significant mass gain and high corrosion rates, reaching up to 74.89 mpy, particularly at higher temperatures near 300 °C.

The highest corrosion rate was for the 5Cr-1/2Mo/ASTM A335GP5 evaluated in crude A and crude B at a temperature of 200 °C, although it generally showed moderate corrosion rates (2.04–5.57 mpy).

The smallest corrosion rate was for X6CrNiMoTi17122/AISI-SAE 316Ti. This shows a straight line with a slight slope for crude A and crude B for the three temperatures (200 °C, 250 °C and 300 °C), demonstrating a superior resistance to sulfidation corrosion by showing the lowest corrosion rate (0.2–0.87 mpy). It makes this a promising candidate for applications demanding high corrosion resistance.

The results show that the corrosion rate (mpy) augments with increasing temperature (°C) for the three materials, 5Cr-1/2Mo/ASTM A335GP5, X6CrNiMoTi17122/AISI-SAE 316Ti and Ni66.5Cu31.5/Monel 400, evaluated in crude A and crude B for three temperatures (200 °C, 250 °C and 300 °C). In the case of 5Cr-1/2Mo/ASTM A335GP5 steel, the graph shows that the corrosion rate increases gradually with temperature (200–300 °C) for heavy crude oils A and B.

Concerning X6CrNiMoTi17122/AISI-SAE 316Ti, the chart describes a slight increase in the corrosion rate with temperature (200–300 °C) for heavy crude oils A and B. The corrosion rates for crude A were greater than those for crude B for the three temperatures.

The highest increase in corrosion rate at temperature 200–300 °C was for Ni66.5Cu31.5/Monel 400, with a dramatic increase at 300 °C for crude A and crude B.

Table 4 provides information on corrosion rate (mpy) results by weight lost for the three materials, 5Cr-1/2Mo/ASTM A335GP5, X6CrNiMoTi17122/AISI-SAE 316Ti and Ni66.5Cu31.5/Monel 400, evaluated in crude A and crude B at three temperatures (200 °C, 250 °C and 300 °C), after a 96 h corrosion test under dynamic conditions (600 rpm) at the same conditions of pressure (200 psi). The highest corrosion rates for crudes A and B were for Ni66.5Cu31.5/Monel 400, indicating a high severity. In second place was 5Cr-1/2Mo/ASTM A335GP5 steel and in third place, indicating low severity, was X6CrNiMoTi17122/AISI-SAE 316Ti steel for both crudes A and B.

### 3.2. Surface Morphology Composition

Morphology evaluations of the external surface of corrosion coupons by visual inspection were as follows:

Temperature 200 °C:

- Crude oil A:
- Ni66.5Cu31.5/Monel 400 alloy coupon: Light layer observed.
- 5Cr-1/2Mo/ASTM A335GP5 steel coupon: Light discontinuous layer observed.
- X6CrNiMoTi17122/AISI-SAE 316Ti steel coupon: No layer observed.
- Crude oil B:
- Ni66.5Cu31.5/Monel 400 alloy coupon: Light discontinuous layer observed.
- 5Cr-1/2Mo/ASTM A335GP5 steel coupon: Light discontinuous layer observed.
- X6CrNiMoTi17122/AISI-SAE 316Ti coupon: No layer observed.

Temperature 250 °C:

- Crude oil A:
- Ni66.5Cu31.5/Monel 400 alloy coupon: Light layer observed.
- 5Cr-1/2Mo/ASTM A335GP5 steel coupon: Light discontinuous layer observed.
- X6CrNiMoTi17122/AISI-SAE 316Ti steel coupon: No layer observed.
- Crude oil B:
- Ni66.5Cu31.5/Monel 400 alloy coupon: Light continuous layer observed.
- 5Cr-1/2Mo/ASTM A335GP5 steel coupon: Light continuous layer observed.
- X6CrNiMoTi17122/AISI-SAE 316Ti steel coupon: No continuous layer observed.

Temperature 300 °C:

- Crude oil A:
- Ni66.5Cu31.5/Monel 400 alloy coupon: Light layer observed.
- 5Cr-1/2Mo/ASTM A335GP5 steel coupon: Light discontinuous layer observed.
- X6CrNiMoTi17122/AISI-SAE 316Ti steel coupon: No layer observed.
- Crude oil B:
- Ni66.5Cu31.5/Monel 400 alloy coupon: Continuous dark scale observed.
- 5Cr-1/2Mo/ASTM A335GP5 steel coupon: Continuous dark scale observed.
- X6CrNiMoTi17122/AISI-SAE 316Ti steel coupon: Light dark scale observed.

**Figure 3:**
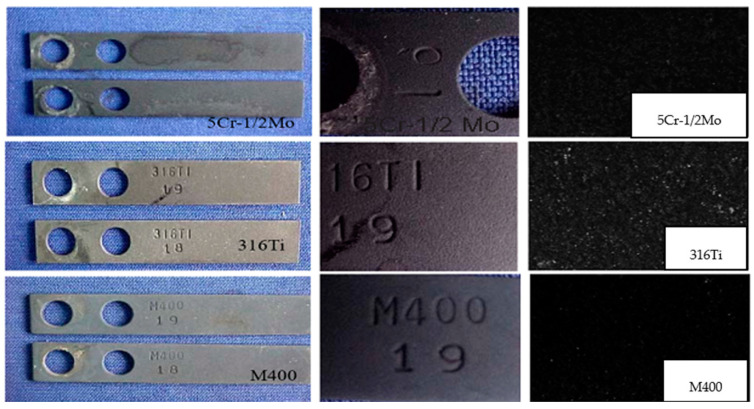
Corrosion coupons: Comparison of corrosion products results.

Table 5 and Figure 3 describe the surface morphology analysis by visual inspection.

Recent studies have demonstrated that high-temperature corrosion by naphthenic acids and sulfidation is influenced by the solid-state diffusion of iron through the inner scale. They proposed that corrosion rates be determined either by chemical kinetics occurring on the surface of the inner scale or by the self-diffusion of iron within the scale itself. A model was developed to simulate this corrosion mechanism and was subsequently validated using experimental data from a flow-through corrosion test [12].

**Figure 4:**
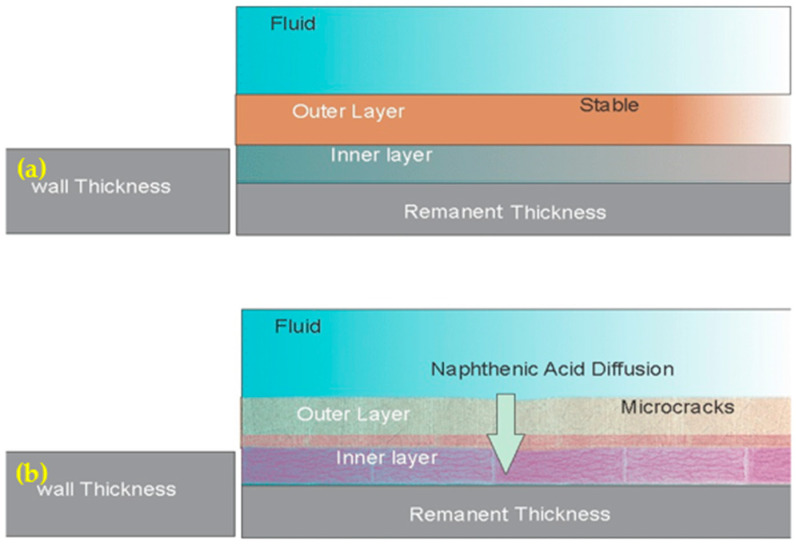
Corrosion coupons: Comparison of corrosion products (**a**) stable layer and (**b**) layer with microcracks.

Figure 4 shows the material loss caused by the reaction between hydrogen sulfide (H_2_S) and steel, resulting in the formation of a sulfide layer. This highlights the crucial role of a stable iron sulfide scale in corrosion protection. As illustrated in Figure 4a, a stable scale can act as a barrier, shielding the metal from further corrosion. However, Figure 4b shows that microporosities within the scale and the presence of naphthenic acid can significantly increase the corrosion rate and damage the metal through the solid-state diffusion of naphthenic acid into the metal and the subsequent formation of iron naphthenate.

Evaluation of the surface of corrosion coupons for three alloys 5Cr-1/2Mo/ASTM A335GP5, X6CrNiMoTi17122/AISI-SAE 316Ti and Ni66.5Cu31.5/Monel 400 (crude B at 300 °C), showed a black layer (scale). Additionally, the three materials were evaluated by SEM–EDS and the Ni66.5Cu31.5/Monel 400 alloy was evaluated by XRD, as described below.

**Figure 5:**
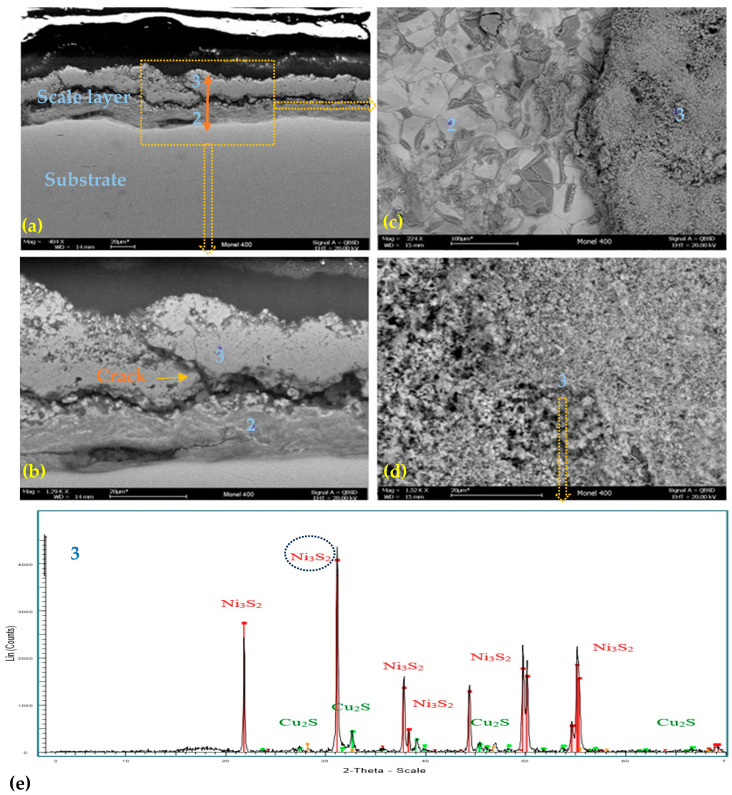
(**a**,**b**) Morphological analysis by SEM–EDS, scale formed on Monel 400 exposed to CRUDE B, at 300 °C, cross section (**a**) 400×, (**b**) 1.29k×; (**c**,**d**) Morphological analysis by SEM–EDS, scale formed on Monel 400 exposed to CRUDE B, at 300 °C, top view (**c**) 224×, (**d**) 1.52k×; (**e**) XRD results of the layer formed on the Ni66.5Cu31.5/Monel 400 exposed to CRUDE B, at 300 °C.

Figure 5 and Table 6 present the SEM–EDS microscopy and DRX results for corrosion deposits or scales on the Ni66.5Cu31.5/Monel 400 coupon for crude B at 300 °C. The EDS results in Table 6 indicate elemental sulfur in the scale on the substrate. Figure 5a,b show a transversal cut (400× and 1.29k×) with uniform layer scales on the metal substrate. Figure 5c,d describe a top view (224×, 1.52k×) of the uniform acicular scale formed on the corrosion coupon. Figure 5d (XRD test results of scale on corrosion coupon) indicates sulfur compounds (Heazlewoodite—Ni_3_S_2_, Copper Sulfide—Cu_2_S), corroborating the EDS results.

Figure 5a,b represent a scale layer with a crack zone. This is a vulnerable area where corrosive agents can diffuse into the substrate, initiating corrosion.

**Figure 6:**
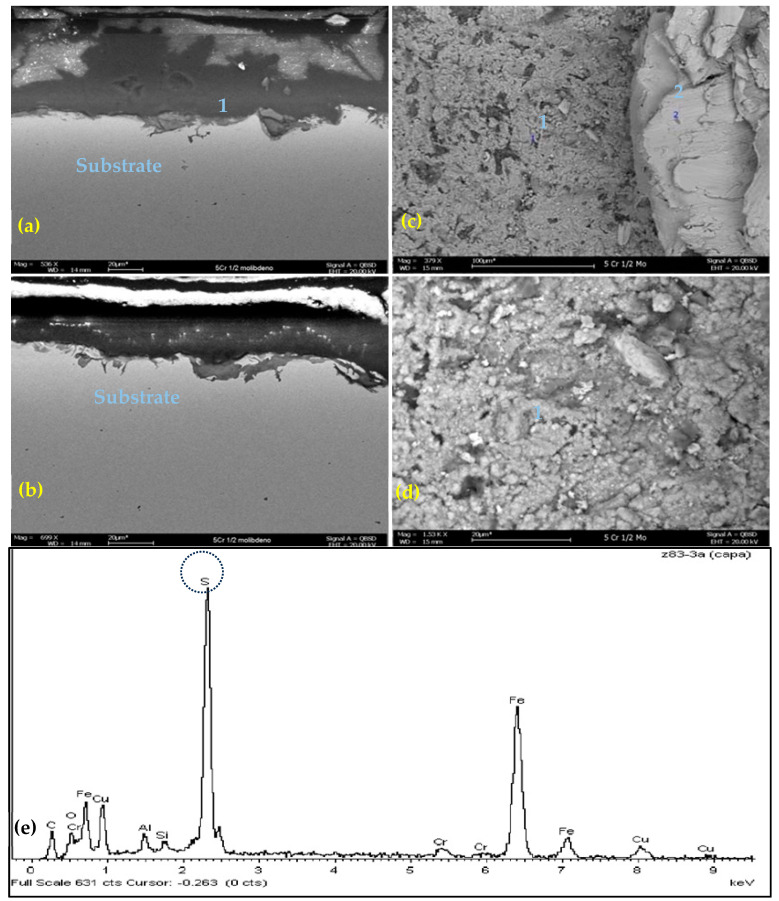
(**a**,**b**) Morphological analysis by SEM–EDS, scale formed on 5Cr-1/2Mo/ASTM A335GP5 exposed to CRUDE B, at 300 °C cross-section (506×); (**c**,**d**) Morphological analysis by SEM–EDS, scale formed on 5Cr-1/2Mo exposed to CRUDE B, at 300 °C, top view (1.53K×); (**e**) Elemental analysis by EDS, layer formed on 5Cr-1/2Mo/ASTM A335GP5 exposed to CRUDE B, at 300 °C.

Figure 6a,b show SEM–EDS microscopy of a cross view of slight layer scale on 5Cr-1/2Mo/ASTM A335GP5 steel corrosion coupons evaluated in crude B at 300 °C. Figure 6c,d illustrate the top view of scale on corrosion coupons. Figure 6d also describes the EDS results, with a peak of elemental sulfur in the scales on the substrate.

Figure 6a,b illustrates a cross-section of the substrate, revealing a discontinuous layer of corrosion products. There was no evidence of cracking or localized corrosion on the coupon surface.

**Figure 7:**
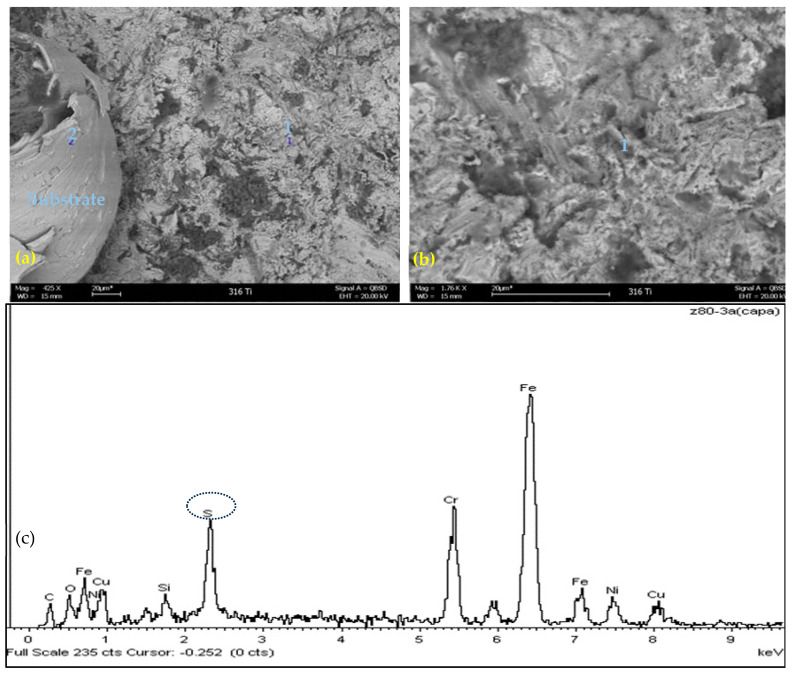
(**a**) Morphological analysis by SEM–EDS, scale formed on 316Ti exposed to Crude B, at 300 °C, top view (425×); (**b**) Elemental analysis by EDS, scale formed on 316Ti exposed to Crude B, at 300 °C, top view (1.76K×); (**c**) Elemental analysis by EDS, layer formed on X6CrNiMoTi17122/AISI-SAE 316Ti exposed to CRUDE B, at 300 °C.

Figure 7a,b present the SEM–EDS microscopy of scale on X6CrNiMoTi17122/AISI-SAE 316Ti, evaluated in crude B at 300 °C, a top view of the corrosion coupons with a small discontinuous scale on the substrate. The EDS test results indicate the presence of elemental sulfur, as shown in Figure 5c.

Figure 7a,b shows the coupon surface with general corrosion and discontinuous corrosion deposits. The surface exhibited slight sulfur deposits. The coupon surface was free from cracking and localized corrosion.

Ni66.5Cu31.5/Monel 400 shows a uniform black layer on the surface for crude B at 300 °C. This was evaluated by SEM–EDS and XRD to corroborate the interaction of metal and corrosive agents. On the other hand, 5Cr-1/2Mo/ASTM A335GP5 shows a discontinuous black layer with elemental sulfur and oxygen peaks. X6CrNiMoTi17122/AISI-SAE 316Ti shows a discontinuous, very slightly black layer in crude B at 300 °C. The SEM–EDS shows elemental sulfur and oxygen peaks, an indication of sulfidation and corrosion.

## 4. Discussion

This study aimed to evaluate the corrosivity of two Colombian heavy crude oils (A and B) on three materials using a dynamic autoclave to simulate refinery conditions. The materials, 5Cr-1/2Mo/ASTM A335GP5, X6CrNiMoTi17122/AISI-SAE 316Ti and Ni66.5Cu31.5/Monel 400, were exposed to the crude oils at various temperatures (200 °C, 250 °C and 300 °C) under controlled conditions of pressure (200 psi) and rotation velocity (600 rpm). The results are promising for applications requiring high corrosion resistance. X6CrNiMoTi17122/AISI-SAE 316Ti demonstrated a low corrosion rate (0.2, 0.87 mpy), making it a strong candidate, while the Ni66.5Cu31.5/Monel 400 exhibited significant corrosion rates up to 74.89 mpy, especially at higher temperatures, and 5Cr-1/2Mo/ASTM A335GP5 generally showed moderate corrosion rates (2.04–5.57 mpy).

The high-temperature corrosion test was performed under dynamic load at 200 psi and 600 rpm for 96 h. The morphology and composition of scales on corrosion coupons for the three materials, 5Cr-1/2Mo/ASTM A335GP5, X6CrNiMoTi17122/AISI-SAE 316Ti and Ni66.5Cu31.5/Monel 400, were evaluated to determine their corrosion resistance in corrosive heavy crude oils A and B. A physicochemical characterization of the corrosion layer was carried out using scanning electron microscopy (SEM–EDS) and X-ray diffraction (XRD) techniques in order to determine its microstructure and elemental composition. However, at high magnifications in SEM analysis, no visually continuous layer was found on the surface of any of the three materials exposed to crude A at any temperature or for crude B at 200 and 250 °C.

For the coupons exposed to crude B at 300 °C, the scale formed on them was evaluated using EDS for three materials. Additionally, XRD analysis was performed on the Ni66.5Cu31.5/Monel 400 alloy to identify the nature of the layer and corroborate the formation of sulfide products. After cleaning, the surface of the corrosion coupons of all three materials, 5Cr-1/2Mo/ASTM A335GP5, X6CrNiMoTi17122/AISI-SAE 316Ti and Ni66.5Cu31.5/Monel 400, exhibited uniform corrosion without evidence of pitting. This is coincident with the SEM–EDS, DRX and corrosion rate results, suggesting a mechanism involving naphthenic acid corrosion and sulfidation interaction.

The X6CrNiMoTi17122/AISI-SAE 316Ti steel exhibited the lowest corrosion rates among the tested materials when exposed to both heavy crude oils A and B within the evaluated temperature range, indicating a pseudo-passive layer resistant to corrosion by naphthenic acids and sulfur compounds. The scale formed on the surface of the coupon and evaluated by SEM–EDS for crude oil B at 300 °C shows traces of sulfur, oxygen and carbon, indicating corrosion due to naphthenic acids and sulfidation interaction.

The Ni66.5Cu31.5/Monel 400 alloy showed the highest corrosion rates for the two heavy crude oils, A and B. In the evaluated temperature range, the corrosion coupon subjected to crude oil B at 300 °C presented a dark-colored layer scale, which was analyzed by DRX and EDS and acicular morphology evidenced the presence of nickel and copper sulfides (Heazlewoodite—Ni_3_S_2_, Copper Sulfide—Cu_2_S), indicating that sulfidation was the predominant effect, as Cu and Ni are elements with a tendency to react with sulfur.

The 5Cr-1/2Mo/ASTM A335GP5 steel material exhibited the second highest corrosion rates for the two heavy crude oils, A and B, in the evaluated temperature range. The chemical composition shown by EDS at 300 °C in crude oil B confirms the presence of sulfur, oxygen and iron, indicating an interaction between naphthenic corrosion and sulfidation, a typical characteristic of sulfidation accelerated by naphthenic acids [18].

The 3xx Series stainless steels (Type 304, 321, 347) are also corrosion-resistant to high-temperature H_2_S and are widely used in preference to 12 Cr for cyclones because of their superior room temperature mechanical properties following periods of extended high temperature exposure [19].

Elevated temperature sulfidation severity depends on total sulfur and naphthenic acid (above 232 °C/450 °F) and the presence of either free sulfur (S) and hydrogen sulfide (H_2_S) or H_2_S and hydrogen (H_2_). Naphthenic acid and sulfide can synergistically increase corrosion rates [20]. In environments containing both hydrogen (H_2_) and hydrogen sulfide (H_2_S), sulfidic corrosion becomes more pronounced. Additionally, naphthenic acid corrosion (NAC) can be inhibited by the formation of sulfide scales in the presence of H_2_S. These complex interactions significantly impact corrosion behavior, potentially leading to increased or decreased corrosion rates. It is crucial to note that sulfidation and hydrogen attack pose severe risks, as they can cause catastrophic pipe failures, resulting in the release of flammable substances, fires, explosions and personal injury [6].

According to the scale formation mechanism, both sulfidation and naphthenic acid (NAP) corrosion involve the movement of iron away from the steel surface. Reactive sulfur compounds quickly form a dense iron sulfide scale through a direct reaction near the surface, growing at a logarithmic rate. This is followed by the slower linear growth of an outer layer, controlled by the diffusion of iron through the inner layer [12].

In general, carbon steels and low-alloy steels up to (and including) 9Cr-1Mo can be susceptible to H_2_ free sulfidation corrosion. Below 450 °F (230 °C), the corrosion rate is negligible. Between 450 °F (230 °C) and 500 °F (260 °C), refinery operating experience has shown that sulfidation rates are normally extremely low (typically less than 1 mpy). The corrosion rate increases with temperature from 500 °F (260 °C) to approximately 800 °F (425 °C). The increase is usually more exponential in nature than linear. Furthermore, the corrosion rate is believed to peak around 800 °F (425 °C), and the rate tends to decrease at higher temperatures. This temperature-dependent behavior may explain why relatively low sulfidation rates are often observed in some of the hottest H_2_S-containing sections of FCC units. There are numerous conjectures as to why there is a peak in corrosion rate, including coke formation, destruction of reactive sulfur species, and the formation of iron sulfide scales that exhibit more stable behavior at elevated temperatures [21].

The iron sulfide film formed as a result of sulfur attack is protective, but beyond a certain velocity and temperature, the film breaks down, resulting in accelerated corrosion. Quite early in the twentieth century, it was found that by alloying with chromium, resistance to high-temperature sulfur attack of iron can be increased [22].

The sulfidation scales formed on metal surfaces act as protective barriers against acid corrosion. However, naphthenic acid (NAP) can still reach the metal by diffusing through cracks or pores in the scale structure. This allows the acid to continue corroding the metal and dissolve the scale itself, reducing its protective properties and increasing the damaging effects of NAP acid [23].

The nature, thickness and morphology of the FeS films, which provide protective properties to steels, are influenced by temperature and exposure time. The protective effectiveness of the outer iron sulfide layer depends on the balance between iron sulfide precipitation, which forms the layer, and corrosion, which can undermine it. According to gravimetric evaluations conducted under simulated transfer line conditions for a high-sulfur heavy crude oil, at temperatures ranging from 250 to 330 °C and exposure times between 36 and 60 h, the corrosion rate of AISI-316 steel continuously decreased due to the formation of a thermodynamically stable troilite film on the material surface [24].

## 5. Conclusions and Future Work

This study evaluated the performance of three materials, 5Cr-1/2Mo/ASTM A335GP5, X6CrNiMoTi17122/AISI-SAE 316Ti and Ni66.5Cu31.5/Monel 400, when exposed to two Colombian heavy crude oils (A and B) under controlled conditions. Testing was conducted at varying temperatures (200 °C, 250 °C and 300 °C) with constant pressure (200 psi) and rotation velocity (600 rpm) for 96 h.

A critical interaction between sulfidation and naphthenic acid corrosion was observed, highlighting the importance of materials’ corrosion resistance in the oil and gas industry.

This research highlights the limitations of using TAN and sulfide percentage as standalone predictors of crude oil corrosivity. Despite low TAN values, substantial corrosion was observed. Additionally, the protective properties of iron sulfide scale were not directly correlated with its thickness or morphology in preventing naphthenic acid corrosion.

The results indicate that sulfidation began at surprisingly low temperatures, around 200 °C. As temperatures rose above 250 °C, sulfidation accelerated significantly. This is likely due to the decomposition of sulfides at higher temperatures, which releases hydrogen sulfide (H_2_S) and forms iron sulfide corrosion products.

The stability of the sulfide scale is crucial for corrosion protection. For instance, a stable scale could act as a barrier, shielding the metal from further corrosion. Nevertheless, microporosity in the scale and naphthenic acid presence can significantly increase the corrosion rate and damage the metal.

Among the tested materials, X6CrNiMoTi17122/AISI-SAE 316Ti stainless steel demonstrated superior resistance to sulfidation corrosion, exhibiting the lowest corrosion rate (0.2–0.87 mpy). This makes it a promising candidate for applications demanding high corrosion resistance. 5Cr-1/2Mo/ASTM A335GP5 generally showed moderate corrosion rates (2.04–5.57 mpy), while Ni66.5Cu31.5/Monel 400 experienced significant mass gain and high corrosion rates, reaching up to 74.89 mpy, particularly at higher temperatures near 300 °C.

The conclusions extracted from this work are summarized as follows:

- X6CrNiMoTi17122/AISI-SAE 316Ti steel was the material with the lowest corrosion rates (mpy) for the two heavy crude oils A and B evaluated under test conditions, indicating corrosion due to naphthenic acids and sulfidation interaction.
- Ni66.5Cu31.5/Monel 400 alloy exhibited the highest corrosion rates for the two heavy crude oils, A and B, in the evaluated temperature range. For the temperature of 300 °C, evaluated in crude oil B, SEM, EDS and DRX confirmed the dominant corrosion mechanism as sulfidation. This corrosion phenomenon increased with temperature due to an increase in the kinetic energy of the species present in the crude oil that diffused toward the surface of the metal.
- Scales can function as a protective barrier against naphthenic acid corrosion if they are adherent and continuous.
- 5Cr-1/2Mo/ASTM A335GP5 steel material exhibited the second highest corrosion rates for the two heavy crude oils, A and B, in the evaluated temperature range. According to the obtained results, accelerated degradation occurred between 250 °C and 300 °C by the synergistic effect of naphthenic acid and sulfide corrosion. In addition, alloy degradation exhibited a polynomial relationship.
- The variable with the greatest impact on the corrosion rate is the temperature, with the corrosion rate increasing with temperature. This is due to the decrease in the viscosity of the crude oil, which allows a greater rate of diffusion of the particles of the reactive species present in the fluid toward the surface of the materials evaluated.
- The type of corrosion observed on the coupons exposed to crude oils A and B at high temperatures was uniform corrosion. A homogeneous loss of material was observed without erosive or pitting processes for all the evaluated coupons.

This methodology could be applied in the future for other types of alloys and coatings. In addition, this research will continue to investigate hydrogen barrier coatings for hydrogen sulfide (H_2_S) media.

## Figures and Tables

**Table 1:** Experimental matrix conditions for 5Cr-1/2 Mo, 316 Ti and M400 materials.

Material	Temperature (°C)	TAN (mg KOH/g)		Sulfur (wt.%)		Pressure (psi)	Stirred Velocity (rpm)	Time (Hours)
		A ^1^	B ^1^	A ^1^	B ^1^			
5Cr-1/2Mo	200, 250, 300	7.7	0.1	1.5	2.3	200	600	96
X6CrNiMoTi17122	200, 250, 300	7.7	0.1	1.5	2.3	200	600	96
Ni66.5Cu31.5	200, 250, 300	7.7	0.1	1.5	2.3	200	600	96

^1^ Crude A, Crude B.

**Table 2:** Analysis of crude oil composition.

	Crude A	Crude B	
Test	Result	Result	Unit
API Gravity at 15.6 °C (60 °F)	11.3	12.8	°API
Density at 15.0 °C	990	980	kg/m^3^
Sulfur Content	1.552	2.358	% m
Neutralization Number	7.77	0.15	mg KOH/g
Pour Point	18	6	°C
Vapor Pressure	0.41	2.42	psi
Salt Content	3.04	0.06	lb/1000 Bls

**Table 3:** Materials composition.

Element	5Cr-1/2Mo/ASTM A335GP5 (%)	X6CrNiMoTi17122/AISI-SAE 316Ti (%)	Ni66.5Cu31.5/Monel 400 (%)
Carbon	0.15	0.08	0.2
Silicon	0.4	0.75	0.5
Sulfur	0.03	0.02	0.023
Phosphorus	0.03	0.45	-
Chromium	5	18	-
Manganese	0.5	2	2
Nickel	-	12	Balance
Molybdenum	0.45	3	-
Titanium	-	0.8	-
Cupper	-	-	28
Iron	Balance	Balance	2

**Table 4:** Corrosion rate vs. temperature results at 600 rpm, test time (96 h).

Material	Temperature (°C)	Crude	Weight Lost (g)	Corrosion Rate (mm/y)
5Cr-1/2 Mo, (ASTM A335GP5)	200	A	0.0134	3.40
	200	B	0.0181	2.04
	250	A	0.0142	3.63
	250	B	0.0134	3.37
	300	A	0.0219	5.57
	300	B	0.0079	4.62
M400, (Ni66.5Cu31.5)	200	A	0.0095	2.19
	200	B	0.0046	1.06
	250	A	0.0269	6.10
	250	B	0.0374	8.47
	300	A	0.1848	42.38
	300	B	0.3247	74.89
SS 316 Ti, (X6CrNiMoTi17122)	200	A	0.0024	0.60
	200	B	0.0008	0.20
	250	A	0.003	0.75
	250	B	0.003	0.74
	300	A	0.0035	0.87
	300	B	0.0032	0.81

**Table 5:** Surface morphology analysis by visual inspection.

Temperature (°C)	Crude Oil	Coupon Material	Observation
200	A	Ni66.5Cu31.5/Monel 400	Light layer
200	A	5Cr-1/2Mo/ASTM A335GP5	Light, discontinuous layer
200	A	X6CrNiMoTi17122/AISI-SAE 316Ti	No layer observed
200	B	Ni66.5Cu31.5/Monel 400	Light, discontinuous layer
200	B	5Cr-1/2Mo/ASTM A335GP5	Light, discontinuous layer
200	B	X6CrNiMoTi17122/AISI-SAE 316Ti	No layer observed
250	A	Ni66.5Cu31.5/Monel 400	Light layer
250	A	5Cr-1/2Mo/ASTM A335GP5	Light, discontinuous layer
250	A	X6CrNiMoTi17122/AISI-SAE 316Ti	No layer observed
250	B	Ni66.5Cu31.5/Monel 400	Light, continuous layer
250	B	5Cr-1/2Mo/ASTM A335GP5	Light, continuous layer
250	B	X6CrNiMoTi17122/AISI-SAE 316Ti	No continuous layer observed
300	A	Ni66.5Cu31.5/Monel 400	Light layer
300	A	5Cr-1/2Mo/ASTM A335GP5	Light, discontinuous layer
300	A	X6CrNiMoTi17122/AISI-SAE 316Ti	No layer observed
300	B	Ni66.5Cu31.5/Monel 400	Continuous dark scale
300	B	5Cr-1/2Mo/ASTM A335GP5	Continuous dark scale

**Table 6:** Elemental analysis by EDS, scale formed on Ni66.5Cu31.5/Monel 400 exposed to Crude B, at 300 °C.

Element	Weight%	Atom%
C	9.50	29.15
O	1.84	4.23
Cu	17.03	9.88
**S**	**21.39**	**24.59**
Fe	1.50	0.99
Ni	47.98	30.12

## Data Availability

Data available on request from the corresponding author.
